# Expression and purification of recombinant G protein-coupled receptors: A review

**DOI:** 10.1016/j.pep.2019.105524

**Published:** 2020-03

**Authors:** Daniel N. Wiseman, Abigail Otchere, Jaimin H. Patel, Romez Uddin, Naomi L. Pollock, Sarah J. Routledge, Alice J. Rothnie, Cathy Slack, David R. Poyner, Roslyn M. Bill, Alan D. Goddard

**Affiliations:** a School of Life and Health Sciences, Aston University, Aston Triangle, Birmingham, B4 7ET, UK; b University of Birmingham, Birmingham, B15 2TT, UK

**Keywords:** Expression, Purification, GPCR, SMALP, Review

## Abstract

Given their extensive role in cell signalling, GPCRs are significant drug targets; despite this, many of these receptors have limited or no available prophylaxis. Novel drug design and discovery significantly rely on structure determination, of which GPCRs are typically elusive. Progress has been made thus far to produce sufficient quantity and quality of protein for downstream analysis. As such, this review highlights the systems available for recombinant GPCR expression, with consideration of their advantages and disadvantages, as well as examples of receptors successfully expressed in these systems. Additionally, an overview is given on the use of detergents and the styrene maleic acid (SMA) co-polymer for membrane solubilisation, as well as purification techniques.

## Highlights

• 1713% increase in unique membrane protein structures since 2000.
• Variety of expression methods available to GPCR researchers, now including the eyes of *Drosophila melanogaster.*
• Development of novel solubilisation strategies including the styrene maleic acid co-polymer.

## 1 Introduction

**Fig. 1:**
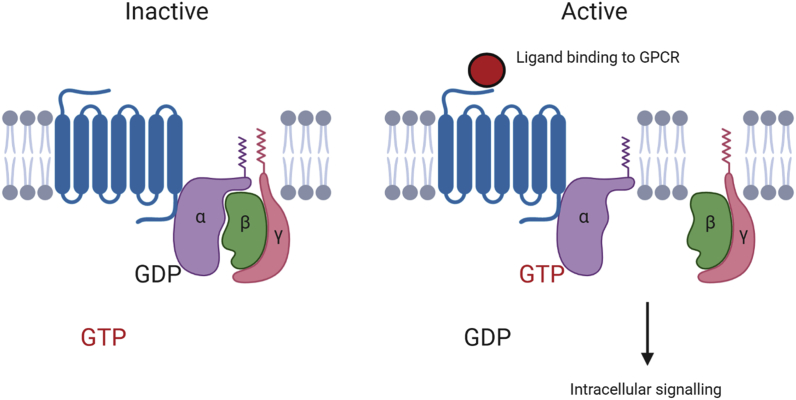
**Ligand induced activation of a G protein-coupled receptor (GPCR).** GPCRs (blue) are transmembrane receptors which activate intracellular signalling pathways, through coupling to G proteins. These heterotrimeric proteins consist of three subunits denoted α, β and γ, and are classically activated by a ligand induced conformational change in the GPCR [2]. This movement is proposed to involve a rotational transmembrane helix reorientation, exposing an intracellular binding cleft [3]. GDP is exchanged for GTP on Gα, while the βγ complex splits away and is able to signal independently of the Gα subunit. Humans encode 18, 5 and 12 different α, β and γ subunits, respectively. These combine into a variety of stimulatory (G_s_) or inhibitory (G_i/q_) effects on pathways including those dependent on adenylyl cyclase and phospholipase C. Created with Biorender.com.

As the largest family of membrane proteins in the human genome, G protein-coupled receptors (GPCRs) are widely studied due to their involvement in normotypical and pathological cell signalling profiles [1]. Characteristically, as shown in Fig. 1, these seven-transmembrane receptors undergo a conformational change upon activation by a ligand, allowing propagation of signalling cascades within the cell [2].

Understanding the relationship between a GPCR's structure and function will aid further development of ortho- and allosteric molecules against these receptors to affect their pharmacology. While approximately half of all drugs target GPCRs, this is only reflected in a 5% coverage of these receptors, providing significant scope for further structure-based novel drug discovery [4].

While the structure of some GPCRs have been successfully determined, many challenges remain in this field. These include the concepts of homo-dimerization, heteromeric protein-protein interactions and the structural complexity of important motifs [5] – of note, the folding and flexibility of the ligand-binding domain of family B GPCRs [6]. While computational biology has greatly enhanced the versatility of studying GPCR structures [7], classical techniques are also often employed including x-ray crystallography [8], cryo-electron microscopy [9] and nuclear magnetic resonance spectroscopy [10]. A significant drawback to these methods lies in the initial requirement for a high yield and purity of mature, folded target protein.

These challenges have propelled the development and implementation of recombinant membrane protein expression, solubilisation and purification systems over the last two decades - contributing, in no small part, to the increase in resolved structures of membrane proteins in the same time-frame [11]. This review will provide a current summary of the methodology, benefits and drawbacks of the expression systems available to GPCR researchers (Table 1), as well as an overview of applicable solubilisation and purification techniques.

**Table 1:**
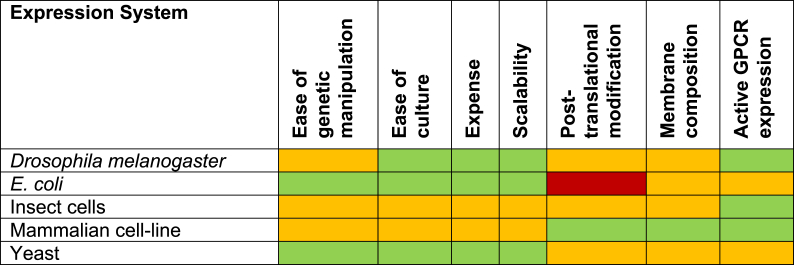
**A comparison of expression systems for GPCRs.** A qualitative assessment of considerations linked to recombinant protein expression systems. Green = positive, amber = moderate, red = negative. While *E. coli* and yeast were historically favourable due to ease of genetic manipulation, culture and scalability, recent developments in insect and mammalian lines have increased their use. Standard expression vectors can now be grown in litre volumes in lines including S*f*9, expi293 and expiCHO at comparable cost to produce milligram quantities of receptor. The use of *Drosophila* is an emerging yet promising method requiring further attention.

## 2 Expression systems

### 2.1 *E. coli*

Native expression of GPCRs is well-known to be restricted to eukaryotic organisms [12] yet *E. coli* has proved an attractive host for expression and purification of a subset of receptors [13]. *E. coli* has become a laboratory workhorse for a number of reasons. Firstly, decades of work have led to well-characterised, rapidly growing cells which are easy to culture and strains have been optimised for protein expression, including membrane protein-specific strains [14]. *E. coli* can be easily grown across a range of scales allowing fermentation to produce large quantities of protein, although it should be noted that there is not always a linear relationship between culture volume and product yield [14]. The genetic tractability of *E. coli* allows a variety of expression plasmids to be used to tune protein expression levels. This can be particularly important with membrane proteins when saturation of the translocon can be a rate-limiting step [13,15]. Indeed, high level expression can lead to formation of inclusion body and refolding of GPCRs from such environments has met with limited success [16]. Some of these problems can be overcome with judicial strain selection and expression at lower temperatures [17]. However, due to its prokaryotic nature, *E. coli* does not possess a number of features that can be essential for GPCR function. There is a lack of post-translational modification, including glycosylation, which can be essential for ligand binding [18]. Despite this, there are several examples of active receptor expression [[19], [20], [21], [22], [23], [24], [25], [26], [27], [28], [29], [30], [31], [32], [33], [34]] including the neurotensin and cannabinoid CB2 receptors. The use of *E. coli* in this sense can also be supported by the ability of deglycosylated receptors [35] to bind ligand, and protein engineering for stability [36]. Additionally, the lipid membrane environment may not include essential components such as cholesterol [37] and contains a very different lipidome to eukaryotic cells – there is clear evidence for lipid-dependent GPCR activity [38].

Despite these clear limitations [39], there have been a number of reports of GPCRs being successfully expressed in *E. coli* [[19], [20], [21], [22], [23], [24], [25], [26], [27], [28], [29], [30], [31], [32], [33], [34]]. Unmodified GPCRs tend to have low stability and may aggregate in such systems [33]. A key strategy for successful expression and correct folding of GPCRs in *E. coli* is the use of fusion partners [40]. These serve both to direct the correct insertion of the receptor into the membrane whilst also increasing its overall solubility, thereby aiding both expression and purification [26]. Additionally, strategies such as selective mutagenesis to introduce stabilising mutations and the use of insertions or truncations has proven successful in some cases [17,33]. Indeed, the genetic tractability of *E. coli* can be used to select for variants with increased stability and expression even for relatively intractable receptors [[41], [42], [43]].

There are a number of advantages of the use of *E. coli* for downstream applications. It is relatively easy to conduct isotopic labelling experiments such that the subsequent protein can be used for NMR studies [10]. It should, however, be noted that the relatively low expression levels of GPCRs in *E. coli* is further impacted by such labelling strategies [10]. However, through optimised expression it has been reported that GPCR expression of up to 50 mg/L can be achieved [40]. The genetic amenability and tools available for *E. coli* open possibilities to select GPCR variants with enhanced expression and stability, generate those “locked” in a particular conformation, and also to, potentially, engineer those with completely novel functions [44]. Despite its prokaryotic nature, *E. coli* has clear potential for at least a subset of GPCRs.

### 2.2 Yeast

The fission yeast *S. pombe* and baker's yeast *S. cerevisiae* are important tools to express and investigate the signalling and stability of GPCRs [[45], [46], [47], [48], [49], [50], [51]], however, the methylotrophic yeast, *Pichia pastoris* (reclassified as *Komagataella phaffii)*, is favored for the overexpression of GPCRs for structural studies [52]. High yields of functional receptors have been expressed [39], including the adenosine 2a receptor [[53], [54], [55]], 5HT5A receptor, beta2-adrenergic receptor [56] and muscarinic acetylcholine receptor M2 subtype (CHRM2) [57]. In addition, high-resolution crystal structures of the histamine H_1_ receptor [58] and the adenosine 2a receptor in complex with an antibody Fab fragment [59] have been obtained using the *P. pastoris* expression system as well as other membrane proteins.

This has been feasible due to the ease of manipulation and stable integration of expression vectors into *P. pastoris* coupled with its ability to grow to high cell densities on glycerol and to utilize methanol as the sole carbon source [60]. This system allows high levels of protein expression to be induced under the tightly controlled *AOX1* promoter [61,62]. Other promoters are also available, including the constitutive glyceraldehyde-3-phosphate dehydrogenase (GAP) promoter as well as emerging novel methanol-inducible, non-methanol inducible and constitutive promoters [63,64]. Several expression vectors and strains are commercially available to optimize protein expression. Commonly used vectors are the pPIC9K, pPICZ or pPICZalpha, the latter of which contains an α-MF signal sequence derived from *S. cerevisiae* to enhance protein secretion. Expression vectors generally contain geneticin, kanamycin or zeocin resistance genes, and auxotrophic markers have also been used for GPCR expression [52,60]. Strains frequently used include the wild type X-33 strain, protease deficient strains (SMD1163, SMD1168 and SMD1165) and auxotrophic strains GS115 and KM71 [11,52,60].

*P. pastoris* is able to perform post translational modifications such as disulphide bond formation, and N and O-linked glycosylation. N-linked glycosylation occurs at the Asn-X-Ser/Thr motif on extracellular domains of GPCRs [65], and in some cases is required for cell surface expression [66], ligand binding and cell signalling [67]. While early steps of *P. pastoris* N-linked glycosylation are similar to the process in mammalian cells [68], it may potentially hypermannosylate the protein which can lead to misfolding, although this is less extensive than in *S. cerevisiae* [69]. It may also glycosylate residues of protein where this would not naturally occur [69,70]. Some GPCRs expressed in *P. pastoris* have therefore been engineered with these sites removed to facilitate crystallisation [58,59]. A study by Yurugi-Kobayashi et al., 2009 [71] which analysed glycosylation-deficient GPCRs demonstrated that while some receptors were expressed with lower functional levels, others were expressed at levels suitable for structural studies when this approach was combined with culture optimisation. *P. pastoris* strains and vectors with humanized N-glycosylation have been developed [68,72], but have not as of yet been applied to GPCR expression. Other modifications to GPCRs expressed by *P. pastoris* include codon optimisation, N and C-terminal truncations [54,59] and T4-lysozyme fusion to intracellular loop 3 (ICL3) [58].

In contrast to mammalian cells, yeast membranes contain ergosterol rather than cholesterol. Membrane cholesterol is thought to be required for the correct function of some GPCRs, and may cause direct conformational changes or indirectly alter membrane properties. Crystal structures suggest that some GPCRs contain specific cholesterol binding sites [[73], [74], [75]]. A humanized *P. pastoris* strain has been engineered which synthesizes cholesterol [76], and could be of benefit for GPCR expression. Cholesteryl hemi-succinate, a cholesterol derivative, can also be added to maintain stability [35, 55, 58].

In summary, yeast possess several advantages over other expression systems, including the ability to perform eukaryotic post-translational modifications while being capable of rapid growth to high cell densities on a large scale in relatively cheap media [77]; these aspects make yeast an appealing host, and their use for GPCR expression has significantly improved knowledge of GPCR structure and function.

### 2.3 Insect cell-line (*Sf*9, *Sf*21, Hi5)

In GPCR structural studies, insect cells are the most commonly used expression system to achieve milligram quantities of protein [35,78]. Expression is achieved via infection with a recombinant form of *Autographica californica*; a multiple nuclear polyhedrosis virus. This baculovirus infects the cells and drives the production of the protein of interest, usually via the polyhedrin promoter [79]. The majority of GPCR studies use the *Spodoptera frugiperda* (*Sf*9) or *Sf*21 cell-line, in preference to *Trichoplusia ni* (High Five) cells [78]. However protein expression levels can vary between different cell-lines so screening of cell lines is necessary when using insect cells for structural studies. The advantages of insect cell expression include growth in serum-free shaker cultures, which decreases costs and enables relatively easy scale-up, as well as high yield and the ability to perform most post-translational modifications [79].

A range of different systems have been developed to generate the recombinant baculovirus [79]. Once generated the virus requires titration in order to achieve an appropriate multiplicity of infection (MOI); an excess of virus will kill the cells before they can be harvested [79]. This step is often the most difficult as how to directly quantify virus is unclear and often inaccurate. Viral plaque assays are commonly used but take a minimum of two weeks and, often, their accuracy is questionable [79]. Various alternative approaches have been devised, including flow cytometry and qPCR, but cost, time and variability can still be problematic [80]. Many researchers find that using the virus directly to express protein in *Sf*9 cells and quantifying the expression level is quicker and more accurate. However, once the virus has been generated and titrated it can be stored for a number of years at 4 °C and much longer at −80 °C. More virus can also be generated by infecting the cells with a high MOI and collecting the cell culture media five days post-infection [81]. Initially, it can take up to a month to produce enough baculovirus to drive large scale expression however, once the virus has been generated, protein can be expressed within a week.

One potential disadvantage of insect cell expression arises from differences in lipid composition compared to mammalian cells. Insect cell membranes are low in cholesterol, have very high phosphatidylinositol content and no phosphatidylserine [35], and as noted below, protein function is highly dependent on lipid environment [82]. There have also been reports that a proportion of the protein produced can be misfolded [83], or that the lytic pathway of viral infection can cause protein degradation [81]. However, overall it remains one of the key approaches for GPCR overexpression.

### 2.4 Mammalian cell-line (HEK293, COS)

Membrane proteins are often expressed in insect, bacterial or yeast expression systems due to their high protein yield and expression, which is advantageous for structural studies [84]. High resolution human membrane protein structures have been solved from recombinant proteins derived from these sources, but the protein conformation and modifications may differ from a human protein expressed in human cells. To address these issues, mammalian cell-lines capable of expressing a desired protein have been trialled [85]. The selection of a specific cell-line is determined by whether the expression system represents the near-native environment in which the desired protein is endogenously expressed [84]. GPCRs are heavily post-translationally modified, therefore expressing human GPCRs in mammalian cells is often ideal to characterise their function and pharmacology [35]. For structural biology however, post-translational modifications can be detrimental during crystal formation. Glycosylation sites affect formation of ordered crystals due to the flexibility and heterogeneity of glycan residues [35]. This issue can be solved by mutating the N-glycosylation sites, provided that the conformation of the receptor is still stable [35]. Alternatively, the use of the GnTI^−^ line which lacks N-acetylglucosaminyl transferase I activity would possibly enable further control of complex glycans [86].

The native environment in which GPCRs are expressed has an impact on the conformation and pharmacology of these receptors. The phospholipid composition of the native lipid bilayer has an allosteric effect on GPCRs. In the case of the human beta 2 adrenergic receptor in different liposomes, synthetic phosphatidylglycerol stabilised the active conformation of the receptor [82]. Due to variations in lipid composition between cell-lines of different expression systems, choosing a human cell-line that has a similar lipid composition to that of the endogenous GPCR is important for receptor pharmacology. As cholesterol can allosterically modulate GPCRs [87], its replacement with ergosterol in yeast expression systems can be detrimental. In the case of the human μ-opioid GPCR, ergosterol constrain the receptor in an inactive state, whereas cholesterol stabilises the active state [88]. Post-translational modification is important for GPCR function, where mammalian cells have the correct enzymes for phosphorylation and palmitoylation of human GPCRs [35].

Yields of recombinant protein in non-mammalian expression systems are often higher than mammalian expression systems, therefore optimising the GPCR gene construct for expression is an important first step [35]. Note, however, that higher levels of expression does not necessarily correlate with functional expression and a robust assay for ligand binding and/or signalling can be essential in any optimisation process [83]. This may take the form of traditional radioligand or fluorescent binding assay or could employ NanoBRET e.g. using β1AR tagged with NanoLuc at the N terminus [89]. In any case, GPCR gene constructs are often codon optimised for mammalian cell expression [90]. Kozak sequences (GCCACCATGG) and signal peptide sequences can be fused to the 5‘ end of the GPCR construct to enhance protein expression and cell surface delivery [90,91]. Subsequently, the optimised construct can be ligated into a plasmid vector, which can be transfected into mammalian cells transiently, or be used to create stable cell lines [35]. While transient transfections with popular reagents give detectable expression at the 48 h mark (on average), this can be extended. The BacMam technology uses a modified baculovirus to give expression within 4–6 h of transduction, lasting up to 5–14 days [92]. Finally, not only are stable lines more reproducible in terms of expression levels, inducible lines have been shown to improve the correct folding of GPCRs when compared to insect cells [83].

Overall, immortalised mammalian cell-lines are useful to study human GPCRs in their wild-type or mutated form. The immortalised human embryonic kidney 293 (HEK293) can transiently express recombinant proteins and is amongst the most popular human cell-line to use [93,94]. HEK293 cells exist as adherent cells or suspension cells; the latter are grown at a higher density, which is useful for protein production [93]. Table 2 summarises the currently known 3D structures of GPCRs derived from expression in mammalian cell-lines, as of 30/8/19.

**Table 2:** **3D structures of recombinant GPCRs derived from mammalian cell-line expression.** Database query generated with MemProtMD at https://blanco.biomol.uci.edu/mpstruc/Accessed 5/9/19.

GPCR	Organism	Cell-Line	Resolution, Å	PDB Entry
Angiotensin type II receptor	*H. sapiens*	Expi293F	2.90	6DO1
CB_1_ cannabinoid receptor	*H. sapiens*	HEK293F	2.80	5TGZ
Cytomegalovirus US28	*H. sapiens*	HEK	2.89	4XT1
Leukotriene B_4_ receptor	*C. porcellus*	HEK293	3.70	5X33
Rhodopsin	*B. taurus*	Cos	3.40	2J4Y
		HEK293S-GnTI^-^	3.30	4A4M
		HEK293S	2.36	6FK6
		HEK293	4.38	6QNO
	*H. sapiens*	HEK293S	3.30	4ZWJ
	*H. adansoni*	HEK293	2.14	6I9K
Smoothened receptor	*H. sapiens*	HEK293S-GnTI^-^	3.20	5L7D
		HEK293S	3.84	6OT0
	*M. musculus*	HEK293	2.80	6O3C

### 2.5 *Drosophila melanogaster*

Each of the conventional expression systems detailed above are not without their limitations. One of the major drawbacks associated with all of these systems is the build-up of immature proteins in the intracellular membranes caused by the cell's failure to properly fold and transport the mature GPCR to the cell surface. This issue can lead to inadequate yields for structural studies thus limiting our understanding of GPCR structure and function [95,96] and obtaining adequate yields of the mature GPCR often requires optimisation of the expression conditions, increasing cost.

The fruit-fly, *Drosophila melanogaster*, has recently been utilised as an attractive alternative expression system to overcome some of these problems. The system takes advantage of the unique properties and architecture of the fly eye which consists of photoreceptor cells (PRCs) containing membrane stacks called rhabdomeres [97] therefore providing a large surface area for expression and folding of large amounts of membrane-associated proteins [98].

**Fig. 2:**
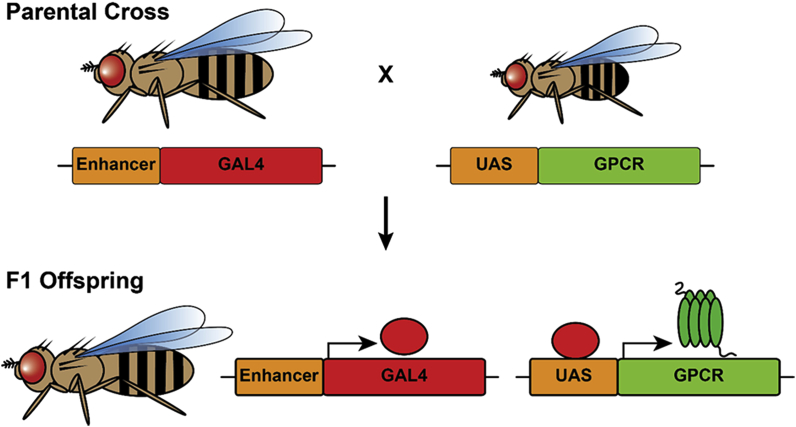
**The *Drosophila* GAL4-UAS system for targeted gene expression.** The GAL4-UAS system can be used for targeted expression of GPCRs within the *Drosophila* photoreceptor cells (PRCs). To obtain flies expressing a gene of interest in a tissue specific pattern, two *Drosophila* strains are mated together in the parental cross. The driver strain expresses the yeast GAL4 protein from a tissue-specific enhancer/promoter. The UAS strain contains the gene of interest cloned downstream of the GAL4 Upstream Activating Sequence (UAS). The resulting F1 offspring will express GAL4 protein in a tissue-restricted pattern which will bind to the UAS sequences upstream of the gene of interest to drive its expression in those specific cells. By using a driver strain that expresses GAL4 specifically within the fly eye, heterologous GPCR expression can be restricted to the PRCs.

Heterologous expression of proteins within the PRCs is achieved using the well-established GAL4-UAS system [99]. This system allows the tissue-specific expression of transgenes by exploiting the use of the yeast GAL4 protein, a transcription factor that specifically binds to an Upstream Activating Sequence (UAS) to drive expression of its target genes. To express a specific protein in a particular tissue type within the fly, two strains are mated together: the driver-strain which expresses GAL4 from a tissue-specific promoter and the UAS strain which contains the transgene of interest cloned downstream of the GAL4 UAS (Fig. 2). In the resulting offspring, the transgene will be expressed in those specific cells that contain GAL4 [99]. By using a driver-strain that specifically expresses GAL4 within the fly eye, heterologous GPCR expression can be restricted to the PRCs. Generating transgenic flies is relatively easy and comparable in cost to other conventional expression systems. Also, *Drosophila* culture media for rearing experimental animals is relatively inexpensive and the need to work in sterile conditions is eliminated when working with flies [98].

*Drosophila* PRCs have been successfully used to express a number of GPCRs [100]; the *Drosophila* metabotropic glutamate receptor, DmGluRA being first reported. Overexpression of DmGluRA in PRCs resulted in higher yields of mature receptor than obtained using other conventional methods, including insect cell culture. Moreover, toxicity effects of DmGluRA overexpression were not observed in the host cells, overcoming a major limitation of other expression systems. Expressing mammalian mGluRs in the fly eye produced similar yields as expressing DmGluRA, suggesting that this system can be used to express foreign GPCRs from other species including human, rat and *Chlamydomonas* [98]. Furthermore, expression of the other two classes of GPCRs have now also been successfully reported using this system [100]. Importantly, it should also be noted that scale-up of expression, as required for downstream processes such as crystallisation, can be readily achieved and can sometimes be an issue for other cell culture-based expression systems [77].

Although the use of *Drosophila* for GPCR expression overcomes several of the major drawbacks associated with more conventional expression systems, it is not without its own limitations. Firstly, this method requires access to fly genetics expertise and facilities for *Drosophila* culture that if unavailable will require the need for collaboration with specialised laboratories that can provide these services [98]. Additionally, although this system is capable of post-translationally modifying proteins there are differences in some of the modifications that occur in the fly that may be important for GPCR function. An example of this is *N-*glycosylation, which tends to be less complex in insects and lacking in extended antennae compared to mammals [101,102]. Furthermore, regarding purification of membrane-associated proteins expressed in the fly eye, there is currently a lack of reports describing the use of detergent-free purification methods such as SMALPs using this system [98,100]. This will be an important future development due to the problems of membrane protein stabilisation associated with using detergents [103,104]. Yet despite these apparent shortfalls, *Drosophila* could prove to be a cheaper and more efficient alternative for functional GPCR expression and purification.

## 3 Solubilisation and purification

### 3.1 Detergents

An important barrier to studying GPCRs is the need to solubilise and purify these membrane proteins away from their native bilayer [105]. Ideally, this process should simultaneously retain target proteins in their folded, functional conformations for further *in vitro* study. Surfactant detergents are able to solubilise and extract membrane proteins due to their amphiphilic nature, improving the aqueous solubility of the protein [106]. A plethora of detergents are commercially available with different physicochemical properties; often, a screen is best performed to identify optimal detergents, likely on a protein-by-protein basis [107].

Briefly, detergents fall into three classes based on their polar head group – ionic, zwitterionic and non-ionic. Ionic detergents such as SDS are regarded as harsh, zwitterions are milder (LDAO) while non-ionic detergents are considered mild. The described harshness is derived from the efficacy of disrupting intra- and inter-molecular interactions. While some factors can be scrutinised, such as the critical micelle concentration (CMC) and hydrophilic-lipophilic balance (HLB), some detergents clearly perform well [108]. Overall, the non-ionic alkyl maltopyranoside detergents DM and DDM have been most successful in contributing to the resolution of membrane protein structures (approximately 45%) [109], and can be regarded as an evidence-based starting point [11,55,58,59,110].

**Fig. 3:**
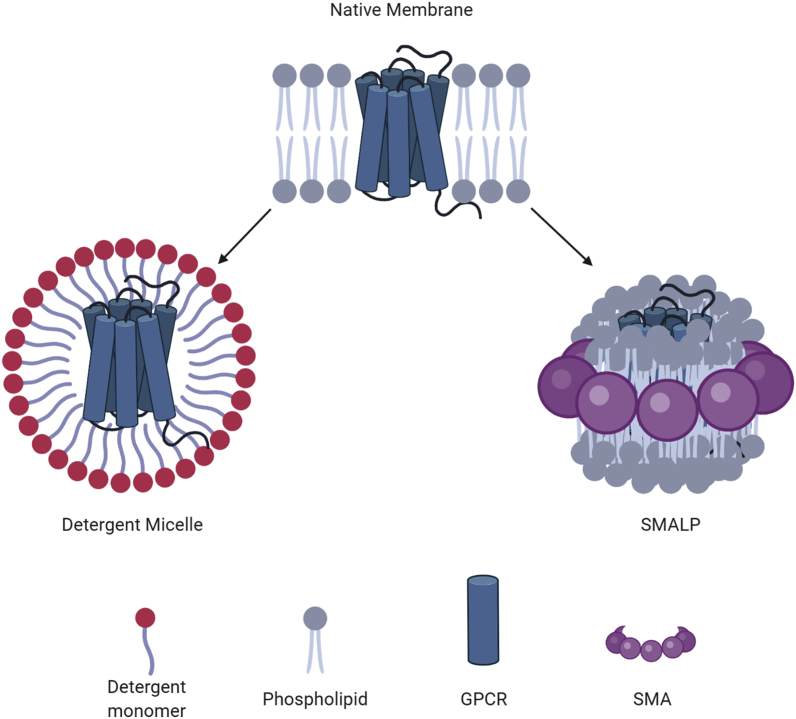
**Solubilised GPCR in a detergent micelle compared to a SMALP.** These diagrams show the interactions of the phospholipid bilayer (grey) or detergent monomers (red) with a GPCR (blue). Importantly, the difference between a detergent micelle and SMALP is shown, with regards to the retention of the GPCR's annular lipids when surrounded by the SMA co-polymer (purple). Created with Biorender.com.

Detergent monomers will, above the CMC, associate with a biological membrane and undergo a transbilayer mechanism to flip from the outer to inner leaflet [111]. This leads to the formation of lipid-detergent micelles (Fig. 3), the efficacy of which depends on the HLB and size/polarity of the detergent molecule. At this point, the bilayer falls apart resulting in the solubilisation of the membrane. A GPCR's protrusion from the bilayer can aid in the incorporation of detergent monomers due to disruptions in the ordered lipid arrangement. However, this can be opposed by the notion of detergent-resistant membranes [112] – especially in regards to GPCR populations in cholesterol-rich lipid rafts [113].

Following solubilisation, it is often necessary to reduce or remove excess detergent to enable purification and further structural or functional analysis. Several methods are sufficient to achieve this including dialysis, size exclusion and affinity chromatography [111].

Despite their utility thus far, detergents are not without their limitations. Understanding of GPCR structure/function has led to the acknowledgement of the native lipid environment. Not only do the lipids surrounding a receptor provide lateral pressure, directly bound lipids can also be essential to influence active/inactive conformations [55]. As such, detergent micelles do not exert the same lateral pressure, and in some cases, the directly bound lipids required for stability/function are removed. It is therefore preferential to adopt techniques which retain these important components to represent a more biologically realistic reflection of GPCRs and their surroundings; and to prevent destabilisation or inactivation during solubilisation [114].

While several new detergents are being designed/developed, other advances reviewed elsewhere include amphipols [115], nano discs [116] and co-polymers including DIBMA [117] and SMA [118]. Largely, detergents must be chosen empirically, which can be an expensive and protein-demanding approach.

### 3.2 SMALPs

GPCRs are one of the classes of protein that have most frequently defied the attempts by biochemists to purify and characterise them. Poor thermal stability is often blamed for this, and considerable effort and resources have been expended to generate thermostabilized versions of GPCRs, in particular for structural studies [119]. An alternative outlook is that GPCRs are destabilised by detergents. Replacing these with better membrane mimetics could prevent destabilisation of the proteins. Amongst the alternatives that have been proposed to meet this need are styrene maleic acid lipid particles (SMALPs). Styrene maleic acid (SMA) is an amphipathic co-polymer that, when added to lipids, spontaneously assembles into nanoparticles of ∼10 nm diameter [120,121]. These nanoparticles consist of SMA polymer surrounding a patch of lipid bilayer (Fig. 3). When SMA is added to biological membranes a similar self-assembly process forms polymer-bound lipid particles containing membrane proteins [[122], [123], [124], [125], [126]].

One of the first reported successes using the SMALP method was the purification and functional characterisation of the adenosine-2A receptor (A_2A_R) which was overexpressed in both human epithelial kidney (HEK) cells and *Pichia pastoris* (Table 3) [55]. This demonstrated that the protein could be rapidly and effectively purified in a detergent-free manner.

**Table 3:** Summary of published methods for the solubilisation of GPCRs by styrene maleic acid and related polymers.

Protein name	GPCR class	Downstream analysis	Source membrane	Polymer used for solubilisation	Reference
A_2a_R	A	Radioligand binding assays, thermal stability	Human endothelial kidney (HEK 293T) cells; *Pichia pastoris*	2:1 SMA (SMA2000P)	[55]
CTR	B	Radioligand binding	Cos cells		[131]
GHS-1aR	A	Ligand binding via FRET; arrestin recruitment; GTP binding assays	Asolectin proteoliposomes	2:1 SMA (SMA2000P)	[129]
HWbR	A	Crystallisation	*Escherichia coli*	3:1 SMA (SMA3000P)	[130]
MT1R	A	Ligand binding; G protein activation; arrestin recruitment	*P. pastoris*	2:1 SMA (SMA2000P)	[129]
V1aR	A	Radioligand binding	HEK 293T	2:1 SMI	[127,131]

The ligand-binding properties of the A_2A_R-SMALPs were used to assay its stability under a variety of conditions. Notably, A_2A_R-SMALPs withstood more than 5 freeze-thaw cycles without reduction in their ligand binding ability. Likewise, A_2A_R-SMALPs and A_2A_R in the membrane both retained 75% of their specific ligand binding capacity after up to 15 days of incubation at 4 °C. In detergent, this binding declined to 0% by day 3. Similarly at 37 °C the stability of A_2A_R in SMALPs far outstripped that of the detergent-solubilised sample. This remarkable stability under a range of conditions makes A_2A_R-SMALPs a much more flexible and useful reagent than the detergent-solubilised equivalent.

A_2A_R was also used in a study of an alternative styrene co-polymer: styrene maleimide (SMI). This has some similar properties and architecture to SMA, but is acid-compatible and can be used in buffers with pH < 7.8 [127]. By contrast SMA is soluble only above pH 5.8, and is more usually used in buffers of pH 8. A_2A_R-SMILPs had ligand-binding properties equivalent to the protein in the cell membrane, indicating that SMI also has potential as a reagent for the detergent-free purification of GPCRs. In the same study, the vasopressin receptor (V_1a_R) also retained its specific ligand-binding properties in SMILPs.

The ability to bind ligands is not the only indicator of GPCR function. Perhaps of more importance is the ability of a purified GPCR to recruit/signal to G-proteins and initiate intracellular signalling cascades [128]. One study using SMA has demonstrated that the melatonin receptor (MT1R) and the ghrelin receptor (GHS-R1a) in SMALPs are capable of G-protein activation, arrestin recruitment and ligand binding [129].

To date, there is only one high resolution structure of a GPCR in SMALPs, *Haloquadratum walsb*yi bacteriorhodopsin (HWbR). This structure was solved at 2.0 Å resolution using the *in meso* crystallisation (lipidic cubic phase) methodology [130]. Hence, it is likely that the protein-SMALPs integrated into the bilayers of the cubic phase prior to crystallogenesis. Lipids are visible in the structure, but these are identifiable as monooleins, the lipids used to assemble the cubic phase. In a parallel experiment, bR purified using detergent had a remarkably similar structure to the structure derived from SMA-solubilised bR. Therefore in this case there is an argument that using SMA did not provide additional structural information compared to using detergent. By contrast, a recent structure was solved by cryo-electron microscopy at 3.4 Å resolution of a bacterial respiratory supercomplex purified using SMA [131]. This structure did show specific native lipids bound to the protein, which may be of relevance in understanding the subtleties of its structure and function. This hints that cryo-EM may be a viable approach for solving structures of membrane proteins retaining their native lipids.

Following solubilisation, several purification methods may be employed which have been reviewed elsewhere [132]. Summarised in Table 4, these include gel filtration, ion exchange and affinity chromatography, of which the latter is most popular. While affinity to antibodies or ligands such as lectin can be utilised for membrane proteins, GPCRs expressed in the systems discussed have utilised a range of purification tags. These include poly-histidine [55,58], FLAG [56,133], HA [134], Strep-Tactin [135], Rho [136] and EF1 [137] tags among others. Following detection and affinity chromatography purification, it is possible to obtain the quantities of functional material required for structural studies with these tags [58,59].

**Table 4:** Pros and cons of purification techniques available for GPCRs.

Purification Technique	Pros	Cons
Affinity chromatography [139,140]	Can be used if protein molecular weight, charge or hydrophobicity is unknown. High affinity binding can result in high sample purity.	May require a tag or terminal fusion. Washing may remove weakly bound molecules. SMALPs are sensitive to divalent cations.
Gel filtration [141,142]	Efficient separation of large and small molecules. Minimal elution volume. No sample loss.	Only separated on size. May require further techniques. Limited resolution due to short chromatogram timescale.
Ion exchange [139]	Only one charge-based interaction. Predictable elution pattern.	Inconsistency between columns. Limited to ionizable groups.

More recent advancements include the use of mini-G proteins to study GPCRs in their active conformations [138]. These are engineered GTPase domains of the Gα subunits of G proteins and stabilise the active conformation of the receptor. Not only have they been shown to form stable complexes purified by SEC, N-terminal fusion with GFP allows for successful detection of coupling by FSEC [138]. Such reagents provide huge potential for state-selective purifications.

## 4 Conclusion

GPCRs remain a challenging component of the membrane protein structural biology field. While the sources of difficulty are gradually being lessened as understanding and technology advance, the dearth of structural information is limiting novel drug design and discovery [4]. Computational biology has greatly enhanced the ability to predict and manipulate GPCR structure, and how this affects their functions. However, *in silico* experiments remain only a component of the holistic study of membrane proteins; expression and purification are largely required before downstream biochemical and biophysical analysis [8,9,143,144].

As such, and discussed in this review, the expression systems available to GPCR researchers each come with their own benefits and drawbacks. While ease of culture and genetic amenability are undoubtedly attractive qualities, they clearly do not entirely make up for biologically important characteristics such as post-translational modifications. There will always seemingly remain a compromise with the expression system of choice, if only the expense. Regardless of these drawbacks, each traditional system will be preferred for application to certain techniques. For example, post-translational modification may be less desired with regards to crystallisation, but more so for trafficking and functionality.

An interesting alternative to consider is cell-free expression [145]. As cell lysate is used, problems such as toxicity and sequestering of protein to inclusion bodies is avoided. Additionally, this technique allows for modification of GPCRs with unnatural amino acids [146] and is a useful method for NMR labelling [10]. Finally, expression in the eyes of *Drosophila* offers a promising solution for a scalable production of functional recombinant membrane proteins (Table 5). Currently, as of September 2019, only five PDB entries were derived from expression in *Drosophila* – none of which were GPCRs. Future work to broaden the diversity and characterisation of varied GPCRs would invaluably reinforce the use of this emerging technique. Overall, the field is currently in a much stronger position than a few decades ago, and will undoubtedly continue to build upon the methods reviewed here.

**Table 5:** **Expression levels of recombinant GPCRs in the photoreceptor cells of *Drosophila melanogaster***. Data obtained from Panneels et al., 2011 [98]. MP = membrane protein.

GPCR	Organism	Expression level, pmol/mg total MP
CCR5 Chemokine receptor	*H. sapiens*	555
DmGluRA Metabotropic glutamate receptor	*D. melanogaster*	226
mGluR5 Metabotropic glutamate receptor	*R. norvegicus*	192
Rh1 Rhodopsin	*D. melanogaster*	502
V2R Vasopressin receptor	*H. sapiens*	>1000
